# Secondary Dentine and Cementum

**Published:** 1921-02

**Authors:** W. Clyde Davis, R. Ottolengui


### SECONDARY DENTINE AND CEMENTUM

BY W. CLYDE DAVIS AND R. OTTOLENGUI

Normal dentin is largely deposited about the dental pulp prior to the eruption of a tooth. But at the time of eruption the pulp canal is very much larger than it will be in adult life, and if the tooth suffer no damage, no line of demarcation will be discernible between the dentin lain down before eruption, and that which is deposited normally thereafter.

In the presence of caries, however, we have long observed the phenomenon to which we have applied the term secondary dentin. As caries approaches the pulp, the pulp is seen to ''contract'' away from the approaching ''enemy.'' Secondary dentin is set up as a barrier, and its microscopic anatomy is so different from normal dentin that any good dental histologist could differentiate between it and normal dentin. There may be some argument as to whether secondary dentin should be accounted as within the realm of physiology or of pathology. The writer is inclined, at least for the present, to look upon it as a physiological occurrence; a normal effort of nature to protect tissue which is attacked by disease. He would not at all confuse secondary dentin with calcification of the pulp, nor with the occurrence of pulp nodules, though even here we are perhaps dealing in technicalities. In chronic arthritis, when the bursae are destroyed, we see an anchylosis of the joint ensuing, much as though nature were arguing, ''If this joint can not be lubricated it must be made immovable.'' By analogy perhaps, when the process of the deposition of secondary dentin as a barrier against approaching caries exhausts the vital resources of the osteoblastic layer of the pulp, some sort of irregular calcification of the organ necessarily ensues. Such calcification, however, so often results in death of the uncalcified terminal of the pulp, with resultant apical infection, that it is not strange that the dental mind should look upon a pulp nodule or a calcified pulp when seen in a radiographic film, as significant of pathology, while at the same time passing over as inconsequential the appearance of deep dippings into the pulp chamber of secondary dentin so often seen under large metal fillings. For our present purpose, therefore, the reader is requested to concentrate his attention upon the fact that true secondary dentin would seem to be a physiological natural defense against an attack of disease.

### SECONDARY CEMENTUM

With this fact so commonly known and so long recognized, it seems strange that we have not heretofore asked whether nature has not a method of natural defense against the death of a tooth pulp. Noyes, Thoma and Grove, and we think Tomes and perhaps others have noted the occasional appearance of cementum which had been laid down after the eruption of the tooth. Grieves several years ago exhibited slides of tooth apices with their foramina closed with cementum and suggested that this was a normal protective process.

### DR. CLYDE DAVIS'S RESEARCH

But we think that the researches of Clyde Davis have carried the investigation to a much more important point, and if we understand his contention, it is that if a pulp be removed aseptically, and without injury to the periapical tissues either by chemicals, trauma or the plantation of bacteria, there is a vivid possibility that the damage will be cured in a perfectly physiological manner by the closure of the foramen with secondary cementum; cementum laid down by the cementoblastic cells of the pericementum in such manner that the foramen will be occluded, and at times even a part of the canal filled with this new cementum.

Dr. Davis's paper before the National will shortly appear in the *Journal* of that association, and we commend its careful study to the profession. We are also promised further papers from him giving full account of his researches, which we shall hope to publish in the near future. Meanwhile, that there may be no misapprehension in regard to Dr. Davis's work and views, the writer takes the liberty of quoting from a private letter recently received, in which Dr. Davis says:

"Please do not confound my work with the consideration of putrescent root canals. That is an issue entirely outside of my research work. I have not found a case where there had been any evidence of putrescence in the apical end of the root that showed any attempt on the part of the vital pulp to close the foramen. * * * The cases I am studying are those where in the radiograph there appears to be no disease whatever."

### RADIOGRAPHIC INTERPRETATION

We must, of course, move forward slowly upon any suggestions for an alteration of root canal technique, lest indeed we move backward. For the immediate present, however, we may permit Dr. Davis's findings to affect our management of pulpless teeth to the following extent.

First, in the past, when we have found tooth roots, especially of elderly persons, occluded with some calcific material near the apices, we have concluded that the canal was blocked with secondary dentin, and as we have been taught that secondary dentin is never found at the actual apex of a root, we have arduously burred and drilled our way toward the supposititious foramen; sometimes we have opened into it, and sometimes we have opened into—elsewhere. In future we must at least consider the possibility that the deposit may be cementum and not dentin.

Second, in the past when we have seen radiographs showing root fillings that did not reach the end we have felt it our duty to remove nice (and often expensive) gold inlays, drill out root fillings and finally attempt to reach the foramen, often with results as described above. We have been made to believe that all such unfilled roots are probably infected, whether bone destruction or radiolucent areas show in the radiographs or not. It is this type of teeth, teeth with roots unfilled, but with no radiographic evidence of periapical disease that inspired Dr. Davis's investigation, and in some hundreds of sections he has found such teeth filled with deposits of secondary cementum.

If we are to found a treatment upon this histological possibility, it is manifest that we must so standardize our radiographic technique that our radiographic readings will be reliable. In future in the absence of systemic disease—let that be repeated for emphasis; in the absence of systemic disease—and in the absence of periapical disease disclosed by the radiograph, and when the radiograph shows a root partially filled, but does not show an unfilled canal beyond, it wil be good treatment to leave the tooth as it is.

If, however, in an exactly similar case the root canal beyond the partial root filling shows as an open passage way, such root should be reopened, cleansed and refilled. It is manifest, therefore, that where the radiograph leads us to believe that the root terminal may be closed with secondary cementum, before adopting this conclusion we should take radiographs at several angles, and they should be good radiographs.

Fortunately, radiographic technique is being so rapidly standardized and improved that films are becoming more and more dependable as a basis for diagnosis. Nevertheless, we can not too strenuously accentuate the point that the radiograph must be a good radiograph, and that it must agree with clinical data and oral examination.—Editorial in the September *Dental Items of Interest.*

